# An Effective Singular Value Selection and Bearing Fault Signal Filtering Diagnosis Method Based on False Nearest Neighbors and Statistical Information Criteria

**DOI:** 10.3390/s18072235

**Published:** 2018-07-11

**Authors:** Zhiqiang Liao, Liuyang Song, Peng Chen, Zhaoyi Guan, Ziye Fang, Ke Li

**Affiliations:** 1 Graduate School of Bioresources, Mie University, 1577 Kurimamachiya-cho, Tsu, Mie 5148507, Japan; zhiqiangliao@126.com (Z.L.); endlesswalts00@163.com (Z.G.); 518M220@m.mie-u.ac.jp (Z.F.); 2 College of Mechanical & Electrical Engineering, Beijing University of Chemical Technology, Chao Yang District, Beijing 100029, China; 3 Jiangsu Key Laboratory of Advanced Food Manufacturing Equipment and Technology, Jiangnan University, 1800 Li Hu Avenue, Wuxi 214122, China; dayanlv@live.cn

**Keywords:** false nearest neighbors, statistical information criteria, selection of effective singular value, low-speed bearing fault diagnosis

## Abstract

Singular value decomposition (SVD) is an effective method used in bearing fault diagnosis. Ideally two important problems should be solved in any diagnosis: one is how to decide the dimension embedding of the trajectory matrix (TM); the other is how to select the singular value (SV) representing the intrinsic information of the bearing condition. In order to solve such problems, this study proposed an effective method to find the optimal TM and SV and perform fault signal filtering based on false nearest neighbors (FNN) and statistical information criteria. First of all, the embedded dimension of the trajectory matrix is determined with the FNN according to the chaos theory. Then the trajectory matrix is subjected to SVD, which is helpful to acquire all the combinations of SV and decomposed signals. According to the similarities of the signal changed back and signal in normal state based on statistical information criteria, the SV representing fault signal can be obtained. The spectrum envelope demodulation method can be used to perform effective analysis on the fault. The effectiveness of the proposed method is verified with simulation signals and low-speed bearing fault signals, and compared with the published SVD-based method and Fast Kurtogram diagnosis method.

## 1. Introduction

Bearings are widely used in rotating machinery, and bearing failures are the most frequent problem. Failures may be catastrophic or may cause major downtime, which will result in production loss and even personal injury or death [1,2], hence, it is significant to analyze and diagnose bearing faults. In bearing fault diagnosis, the fault signal is always non-linear and non-stationary, and the fault signal collected is always submerged in background noise, and the energy distribution of the fault signals is unknown, so it’s hard to make a diagnosis due to the weak energy distribution of fault signals. Therefore, it is of vital significance to carry out effective fault diagnosis on the bearings.

For bearing fault diagnosis, the mainstream methods are to analyze the vibration signals collected from the time domain, frequency domain, time-frequency domain, phase-space dissimilarity measurement and other methods [3,4,5,6,7]. Previous studies indicated that there are mainly two bearing diagnosis methods: one is discriminant model-based, and the other one is fault characteristic frequency-based. The general process of model-based involves collecting the vibration signals in the fault state, and setting up an effective discriminant model for fault diagnosis by means of signal filtering [8] and feature extraction [9]. This method will usually combine some statistics and machine learning methods, such as PCA, CDA, neural networks (NN), support vector machine (SVM) etc. and other intelligent methods [10]. With constant development of deep learning, some methods such as CNN and DBN, etc. are employed to build discriminant models of bearing faults, and good effects are achieved [11,12]. However, in engineering applications, such methods also show their deficiencies: whether the fault can be effectively diagnosed or not depends on the accuracy of the discriminant model, and the accuracy of discriminant model in turn largely depends on the effective extraction of features, and the effective extraction of features is based on good signal filtering. During the whole procedure, if any link of the procedure is not ideal it will seriously affect the fault diagnosis result.

Another fault diagnosis method is a fault characteristic frequency-based method, namely the signal demodulation method. The general process of characteristic frequency-based methods is: filter the acquired signal first, then decompose the signal to extract fault signals or strengthen the fault signals. After that, a demodulation method such as envelope demodulation or morphological demodulation, is used to demodulate the signal to identify the fault characteristic frequencies for fault diagnosis. The signal decomposition is the basis of this method, and the empirical mode decomposition (EMD) is one of the most classical decomposition methods [13,14]. As further studies on decomposition method were carried out, researchers have proposed a series of decomposition methods, such as variational model decomposition [15], intrinsic time-scale decomposition [16], singular value decomposition, singular spectrum decomposition [17,18], multifractal detrended fluctuation analysis [19] etc. Among these decomposition methods, the singular value decomposition (SVD) method is an effective fault diagnosis method. It is a non-linear filtering method, which is able to eliminate random noise components from a signal and obtain a relatively pure fault signal. Additionally, SVD has superior stability and invariability, and the singular value decomposed by it can reflect the intrinsic properties of signals and improve the Signal to Noise Ratio (SNR). It is suitable for fault diagnosis against strong background noise signals. At present, this method has good effectiveness in bearing fault diagnosis [13,17,18]. However, there are two problems not well solved yet: one is how to decide the embedded dimension (length of time window) of the trajectory matrix, and the other is how to choose the best singular value. The embedded dimension is used to construct the trajectory matrix, based on which the SVD, feature recombination and signal restoration are performed; the quality of the embedded dimension affects the final analysis results to a large extent, and currently the selection mainly relies on experience. In SVD fault diagnosis, the improper selection of singular values will significantly influence the final result. The singular value representing background and noise signals is set to zero to achieve the effect of background noise elimination. However, in the example of bearing faults at low-speed rotation, the SNR is low, and the fault signal energy is weak, so the energy distribution of fault signals and background noise are unknown; therefore, it is important to effectively select the singular value. Previously, the singular value selection depended on experiments or trial and error, which always generated relatively large errors. Some studies have elaborated on this problem [20,21,22]. Some researchers tried to seek the singular value by constructing a proper singular spectrum and identifying the turning point. For example, Zhao et al. [23] proposed selecting a singular value using difference spectra. The performance of this method, however, will be reduced against a strong background of noisy signals, as the method mainly focuses on the maximum peak position of the constructed singular spectrum, which may result in the loss of important information about other peaks. Other researchers proposed selecting an effective singular value based on the asymptotic relationship between singular values and vectors of the signal matrix and the observed matrix [20]. The filtered signal matrix is reconstructed by minimizing the asymptotic loss. Its performance is superior to the conventional reduction of singular values accomplished by thresholding methods [24]. However, some assumptions must be met to use this method, such as the orthogonally invariance of the signal noise. The assumption is difficult to satisfy in engineering applications.

In view of the aforementioned issues, the discussed fault methods are not ideal against the background of strong noise. Due to the above problems, this study proposes an effective method to select singular values and applied it in fault diagnosis under low-speed rotation. First of all, according to the reconstruction theory of chaos phase space, the embedded dimension of the trajectory matrix can be reconstructed with the FNN method. The phase space reconstructed with this method can be used to characterize the dynamic features of the motive power system. After the trajectory matrix is determined, the trajectory matrix is subject to SVD, and different SVs acquired from SVD are combined to change the decomposed signals back to one-dimensional signals, which are compared with signals in a normal state; the designed evaluation function of statistical information is used to compare paired signals as well as the similarities between restored signal and signal in normal state. The SV combination with maximum similarities is considered to represent the background signal and noise signal, and the remaining SVs are considered to represent the fault signal. The SV representing the fault signal changes the decomposed signal back to a one-dimensional signal, and effective analysis can be performed on the faults using spectrum envelope modulation. This method has been verified with simulation experiments and engineering experiment, and compared with the published SVD-based method and Fast Kurtogram method to verify the effectiveness of the method.

The remainder of the study is organized as follows: Section 2 describes the mechanism of SVD filtering; Section 3 describes the importance of SV selection for the diagnosis and bearing envelope analysis; Section 4 describes the methods proposed in the study; Section 5 proves the effectiveness of the methods proposed with simulation experiments and engineering experiments, as well as comparison with the published SVD-based method and Fast Kurtogram; Section 6 presents the conclusions.

## 2. Mechanism of SVD Filtering

**Figure 1:**
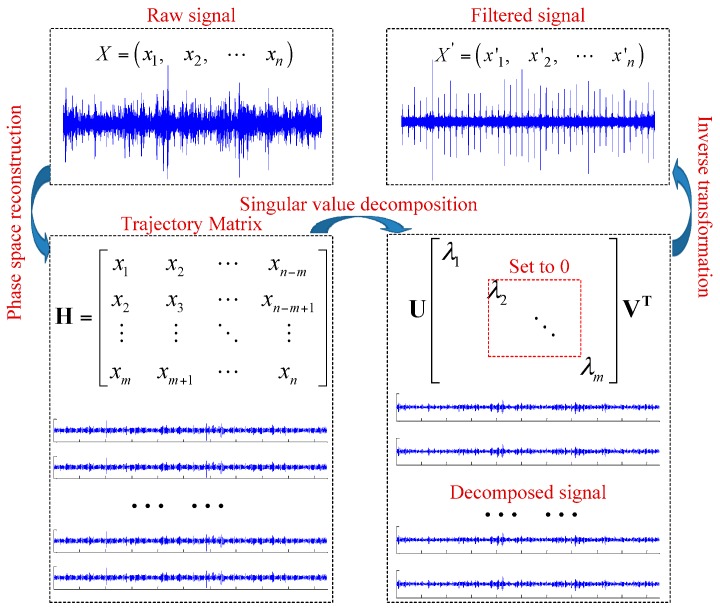
Singular spectrum analysis filtering schematic diagram.

This section illustrates the mechanism of SVD filtering: The signal trajectory matrix is reconstructed based on the embedded dimension obtained from FNN. Then the trajectory matrix is subject to SVD, which is helpful to acquire all the combinations of SV and decomposed signals. SV combination changes the decomposed signal back to one-dimensional signals; if the SV combination can represent the fault signal information, the signal changed back only contains the fault signal information, which is the whole process of signal filtering. A schematic diagram is shown in Figure 1.

The raw signal *X* with noise was measured first. The reconstructed trajectory matrix of a raw signal can be used to characterize the dynamic features of the motive system in the reconstructed phase space. A filter can be designed based on the SVD filtering mechanism to reduce the effect of noise on the reconstructed trajectory matrix of phase space and thus reduce the noise in the observed time series, which corresponds exactly to the SVD filtering mechanism. Based on the SVD filtering mechanism, the background signal and noise signal can be eliminated to generate fault signal shown and identify the fault.

## 3. Description of the Problems in SV Selection and Bearing Envelope Analysis

### 3.1. Bearing Envelope Analysis

Bearing envelope analysis [25] is based on demodulation of high frequency resonance associated with bearing element impacts. For rolling element bearings, impacts are produced when the rolling element strikes the inner or outer race. These impacts modulate a signal at the associated bearing pass frequencies. It is also called fault characteristic frequency. According to [26,27], suppose that *N_b_* is the number of rollers, *D_b_* is the roller diameter, *D_c_* is the bearing pitch diameter. *α* is the contact angle which can be found in the bearing specifications offered by bearing manufacturer. Even if the contact angle is different, when bearing has faults, the signal features are same. *f_f_* is the sampling frequency.

The inner race fault characteristic frequency is *f_I_*, the ball passing frequency on the inner race can be given by:

(1) $$f_{I}=\frac{N_{b}f_{r}}{2}\left(1+\frac{D_{b}}{D_{c}}\cos\alpha \right)$$

The outer race fault characteristic frequency is *f_o_*, the ball passing frequency on the outer race can be given by:

(2) $$f_{O}=\frac{N_{b}f_{r}}{2}\left(1-\frac{D_{b}}{D_{c}}\cos\alpha \right)$$

The roller fault characteristic frequency is *f_B_*, the ball passing frequency on the outer race can be given by:

(3) $$f_{B}=\frac{D_{c}f_{r}}{2D_{b}}\left(1-{\left(\frac{D_{b}}{D_{c}}\cos\alpha \right)}^{2}\right)$$

According to the fault characteristic frequency, it can diagnose bearing faults accurately.

### 3.2. Problems in SV Selection

**Figure 2:**
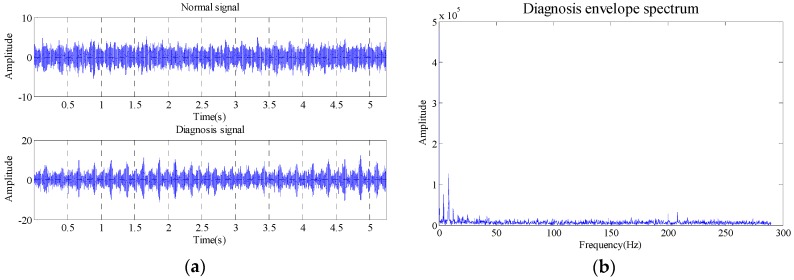
Waveform of time domain and envelope spectrum. (**a**) Time domain waveform; (**b**) Envelope spectrum waveform.

As the energy distribution in background information, intrinsic information about the condition of the bearings and noise information is unknown, and the selection of effective SV has a direct effect on the fault diagnosis at a low rotational speed, medium rotational speed and even a high rotational speed. With the bearing outer race fault experiment as an example, this study elaborates on this problem. The analysis data is from a static load outer race fault experiment. The fault depth: *d* = 0.05 mm; width: *w* = 0.3 mm. The number of rollers: 11; sampling frequency; 50 kHz, rotational speed: 500 rpm. The fault data is measured under above conditions. The rotation rate measured in normal state is 500 rpm. The number of measurements is 262,144 (time: 5.2429 s). The time domain waveform and envelope spectrum of the original signal is shown in Figure 2.

According to the bearing fault characteristic frequency computation formula, the outer race fault characteristic frequency is 36.59 Hz. From the envelope spectrum modulation, this fault characteristic frequency cannot be identified for the diagnosis. For the reconstruction trajectory matrix of this data, the embedded dimension number is set at 8, and it is obtained by FNN method, and the reconstructed trajectory matrix is subject to SVD, and the decomposed SV is as shown in Table 1.

**Figure 3:**
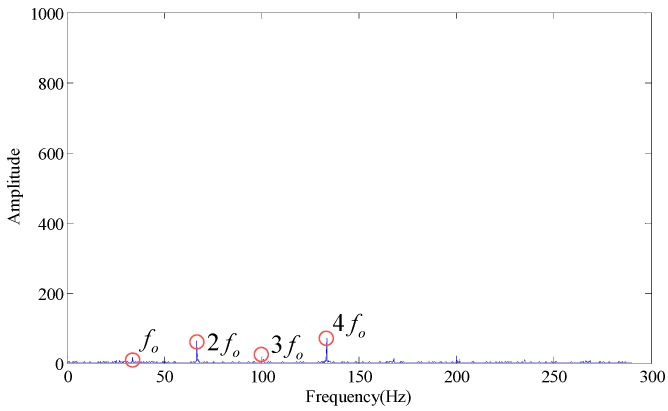
Envelope spectrum (singular values 5~8 are set to 0).

As shown in the Table 1, the maximum SV is much larger than the minimum SV. According to the energy distribution analysis, compared with the first one, the energy of the last few SVs is negligible. According to conventional analysis, the last few SVs are set to zero. According to the singular values difference spectrum method [23], the fifth to eighth SVs are set at zero, and the envelope spectrum obtained is as shown in Figure 3.

**Figure 4:**
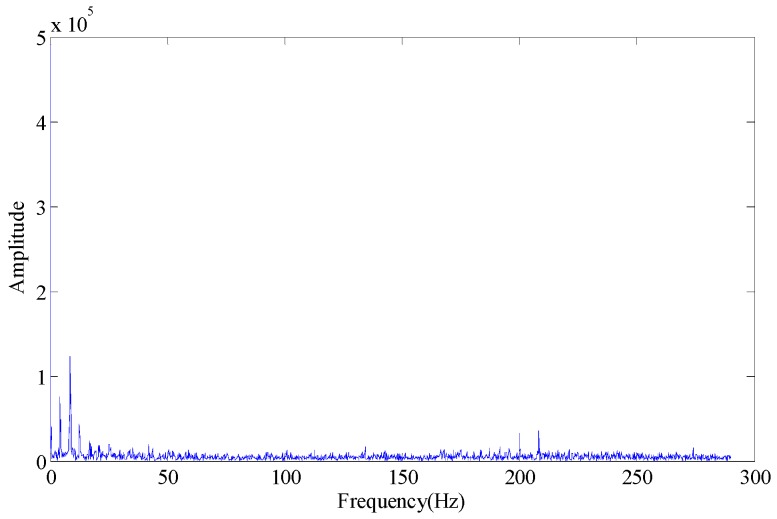
Envelope spectrum (singular values 1~4 set to 0).

**Figure 5:**
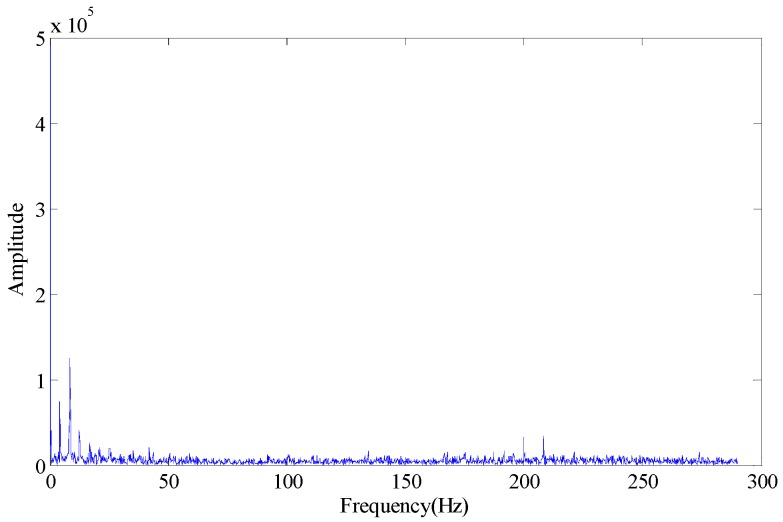
Envelope spectrum (singular values 1, 2, 7, 8 set to 0).

Wherein, *f_o_* is the outer race fault characteristic frequency. According to Figure 3, some trends can be observed in the fault characteristic frequency, but the effect is not obvious. When the first few larger SVs are set at zero (the first SV to fourth SV), the envelope spectrum obtained is as shown in Figure 4. If we set the first two SVs and last two SVs at the same time (the 1st, 2nd, 7th and 8th SV), the effect on the envelope spectrum is as shown in Figure 5.

**Figure 6:**
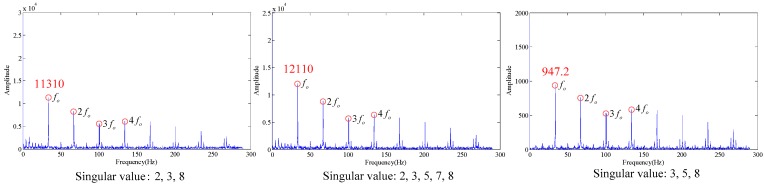
Envelope spectrum under different singular values.

As shown in Figure 4 and Figure 5, the fault characteristic frequency cannot be identified in the envelope spectrum, and the diagnosis fails. It can be seen from the analysis in the presence of the combination of 2, 3, 5, 6, 7 and 8, the effective fault characteristic frequency can be extracted. For example:

It can be seen from the Figure 6 that the above combinations can effectively obtain outer race fault feature frequencies by modulation, and the energy is maximum (12,110) when the SV combination is 2, 3, 5, 6, 7 and 8, so this combination is the combination with optimal filtering effects. It can be seen from the example that the SV selection brings a large effect to the modulation results of rotating bearings.

## 4. Trajectory Reconstruction of Fault Signals Based on Chaos Theory

### 4.1. False Nearest Neighbors

False nearest neighbors [28] is an effective method for calculating the embedding dimension. According to the geometric theory, the chaos time series can be regarded as the projection of chaotic motion of high-dimension phase space on low-dimension space-time. In the projection process, the chaotic motion trajectory will be distorted, and after projection, two original nonadjacent points in high dimensional phase space may become two adjacent points in low-dimension space-time, namely false nearest neighbor points. Phase space reconstruction is a method to recover chaotic motion trajectories from chaotic time series, which can be used to evaluate the existence of nearest neighbor points. Specifically, in *m*-dimensional space, the phase point is *x*(*t*) = {*x*(*t*), *x*(*t* + τ),…, *x*(*t* + (*m* − 1) τ)}, *t =* 1, 2, …, *m*; τ is time delay; each phase point has nearest neighbor point at certain distance *X_f_*, with distance of *R_m_*(*t*) = ‖$X\left(t\right)-X_{f}\left(t\right)$‖. When the dimensional number of phase space is increased from *m* to *m* + 1, the distance between the two phase points will change, and the distance is changed to:

(4) $$R_{m+1}^{2}(t)=R_{m}^{2}+{\left\|X(t+m\tau )-X_{f}(t+m\tau )\right\|}^{2}$$

When there is relatively large change in the ratio of *R_m_*_+1_(*t*) against *R_m_*(*t*), it can be considered as the two neighbor points in high-dimension singular attractor turns into false neighbor points. Make:

(5) $$S_{m}=\frac{\left\|X(t+m\tau )-X_{f}(t+m\tau )\right\|}{R_{m}(t)}$$

If *S_m_* > *S_t_*, *X_f_*(*t*) is the false neighbor point of *X*(*t*). For actually measured time series, when the embedding dimension *m* is increased with the ratio of nearest neighbor point lower than 5% or the false nearest neighbor point no longer decreases with the increase of embedding dimension *m*, it can be considered that the singular attractor is fully unfolded, and *m* is the optimal embedding dimension. 

### 4.2. Signal Trajectory Matrix Reconstruction and SVD

According to the trajectory matrix [29], when the embedding dimension is determined, the trajectory matrix of the restored signal is a Hankel matrix, and the construction of the Hankel matrix is shown as Equation (6):

(6) $$H=\left[\begin{array}{cccc} x_{1} & x_{2} & \cdots & x_{n-m}\\ x_{2} & x_{3} & \cdots & x_{n-m+1}\\ \vdots & \vdots & \ddots & \vdots \\ x_{m} & x_{m+1} & \cdots & x_{n} \end{array}\right]$$

wherein, *n* is the length of experiment data, *m* is the embedding dimension. The SVD of trajectory matrix is as Equation (7):

(7) $$H=U\times \sum \times V^{T}$$

wherein, *U* is a *m* × *m* orthogonal matrices, and *U* × *U^T^* = *I*, *V* is a *n* × *n* orthogonal matrix, and *V* × *V^T^* = *I*; $\sum =\mathrm{diag}\left(\sigma_{1},\sigma_{2},\ldots ,\sigma_{n}\right)$ is a diagonal matrix by descending order, and the diagonal elements are the singular values of *H*.

### 4.3. Statistical Information Criteria

In this paper, the purpose of the statistical information criterion is to select the optimal singular values which can produce the maximum similarity between the rebuilt signal and the normal condition signal. To select and design a reliable criterion, the following two conditions should be taken into consideration:

(1) The similarity of the probability density distribution between the rebuilt signal and normal condition signal should be as high as possible.
(2) The similarity of the frequency density distribution between the rebuilt signal and normal condition signal should be as high as possible.

**Figure 7:**
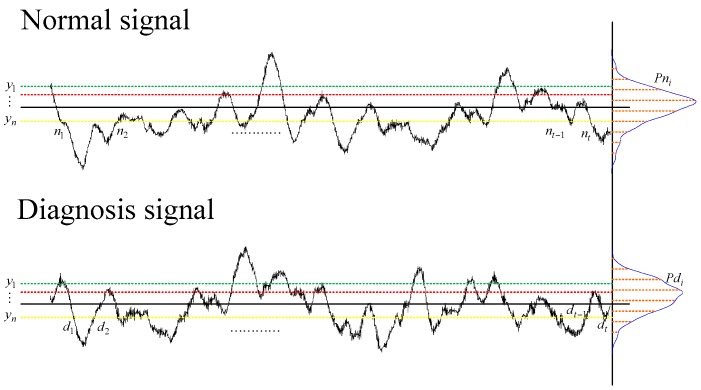
Statistical information criterion schematic diagram.

The statistical information criterion schematic diagram is shown in Figure 7.

The proposed condition requires that the criterion have a strong capability to control the similarity of the probability density distribution and frequency density distribution between the rebuilt signal and the normal condition signal.

For serving the above requirements, a “statistical information criterion” is proposed in this paper. The probability density distribution function can be described as:

(8) $$q_{j}=\frac{1}{\pi }\sqrt{\frac{\int_{0}^{\infty }{\left(2\pi t\right)}^{2}P_{x}\left(t\right)dt}{\int_{0}^{\infty }P_{x}\left(t\right)dt}}e^{-j^{2}/2\sigma^{2}{}_{x}}$$

and the frequency density distribution function can be described as follows:

(9) $$p_{j}=\frac{q_{j}\sqrt{2\pi }}{2\int_{0}^{\infty }{\left(2\pi t\right)}^{2}p_{x}\left(t\right)dt}$$

The statistical information criterion can combine probability density distribution function with frequency density distribution function, and be expressed as:

(10) $$\begin{array}{cc} I_{pq} & =\sum_{j=1}^{n}\left|\log\left\{\left(p_{j}^{*}/p_{j}\right)\times \left(q_{j}^{*}/q_{j}\right)\right\}\right|/n\\ & =\sum_{j=1}^{n}\left|\log\left\{\left(p_{j}^{*}/p_{j}\right)\right\}\right|/n+\sum_{j=1}^{n}\left|\log\left\{\left(q_{j}^{*}/q_{j}\right)\right\}\right|/n\\ & =I_{p}+I_{q} \end{array}$$

where *P_x_*(*t*) is the probability density function value at *t*, $p_{j}^{*}$ is probability density distribution function of the rebuilt signals, *P_j_* is a probability density distribution function of normal condition signal, $q_{j}^{*}$ is a frequency density distribution function of the rebuilt signals, *q_j_* is frequency density distribution function of normal condition signal, *j* from 1 to *n*.

According to the Equation (10), when the value of *I_pq_* is small, it means the similarity is high, the selected singular value combination is considered to represent the background noises information and the rest of the singular value that are out of combination are considered to represent the intrinsic information about the condition of the bearings. The rest of the singular values combined with the decomposed signals for rebuilding the one-dimensional signal can obtain the filtered fault signal.

### 4.4. Procedure of the Proposed Method

**Figure 8:**
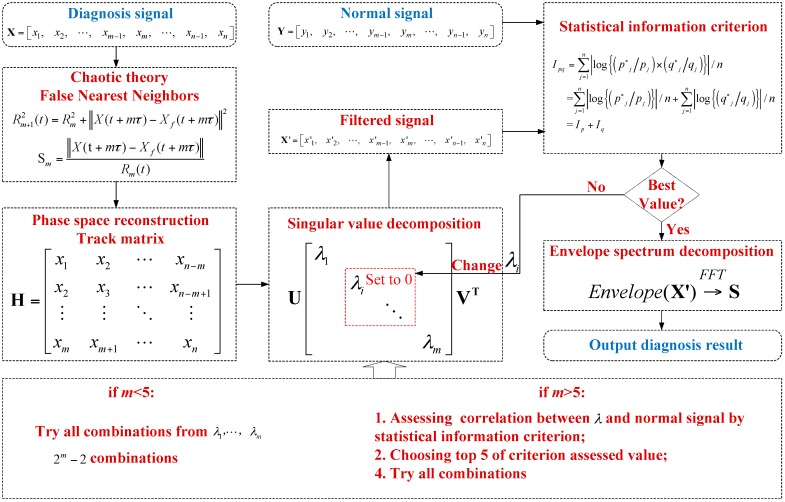
Flowchart of the proposed method.

The signal trajectory matrix is reconstructed according to the false nearest neighbor theory of chaotic phase space reconstruction, and the SVD and statistical information criteria can be used to effectively filter the signals. The procedure is as shown in Figure 8.

Detailed steps of proposed method:

1. Collect abnormal signals and signals in normal state to be diagnosed;
2. Obtain the optimal embedding dimensions from abnormal signals with the false nearest neighbor method;
3. Construct the signal trajectory matrix based on the obtained embedding dimensions;
4. Perform SVD for the trajectory matrix to acquire the corresponding SV;
5. When the number of SVs is less than 5, all combinations are performed for the SVs; when it is higher than 5, the SVs are turned back into one-dimensional signals and compared with that of normal signals, and all combinations are performed for the five SVs with the largest similarities from low to high (the function value evaluated with statistical information is maximum). The number 5 is selected, because there are 30 combinations that should be calculated, and if the number >6 is selected, there are so many combinations, that the efficacy will decrease.
6. The decomposed signals are restored with different combinations and analyzed with the normal signals; evaluation function calculation is performed with statistical information to obtain the similarity of each combination.
7. The SV combination with the maximum similarities is obtained, and the remaining SVs and decomposed signals are turned back into one-dimensional signals, which are demodulated with the envelope spectrum to obtain the fault characteristic frequency and diagnose the fault.

According to the proposed method steps, this is an automatic process which can detect bearing health conditions.

## 5. Experiment and Result Analysis

### 5.1. Simulation Test

**Figure 9:**
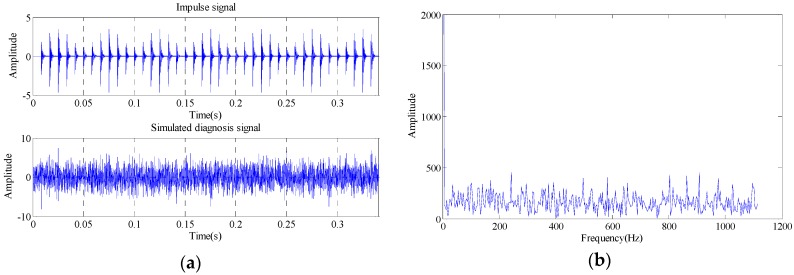
Time domain and envelope spectrum waveform of simulated signal. (**a**) Time domain waveform; (**b**) Envelope spectrum waveform.

In order to verify the effectiveness of the proposed method, an early stage local bearing inner race fault is simulated with the following model:

(11) $$x\left(t\right)=m\left(t\right)\sum_{-\infty }^{\infty }x_{1}\left(t\right)\cdot x_{2}\left(t\right)\cdot x_{3}\left(t\right)+n\left(t\right)$$

(12) $$m\left(t\right)=A(1-B\cos(2\pi f_{sf}t))$$

(13) $$x_{1}\left(t\right)=e^{-c\left(t-kT-\tau_{k}\right)}$$

(14) $$x_{2}\left(t\right)=\cos\left(2\pi f_{n}\left(t-kT-\tau_{k}\right)\right)$$

(15) $$x_{3}\left(t\right)=U\left(t-kT-\tau_{k}\right)$$

k=1,2,3,⋯.

wherein, *m*(*t*) is the amplitude modulation of shock signal (*A* = 4 and *B* = 0.5 in this study). The speed frequency *f_sf_* is set to 20 Hz. Signal damped exponent *C* is 1000, and resonance frequency *f_n_* is 5000 Hz. $\tau_{k}$ is the tiny fluctuation of the *k*-th shock relative to feature cycle *T*, and the random fluctuation complies with standard normal distribution, and the standard deviation is 0.5% of rotational speed frequency, and fault characteristic frequency *f_i_ =* 1/*T* is 120 Hz. *U*(*t*) is heavyside function, and *n*(*t*) is Gaussian noise with standard deviation of 2, and the simulation signal can be obtained by adding the noise signal. Here, sampling frequency *f_s_* is 12,000 Hz, and sampling data length is 4096. The simulated signal waveform figure and envelope spectrum signal are as shown in Figure 9.

**Figure 10:**
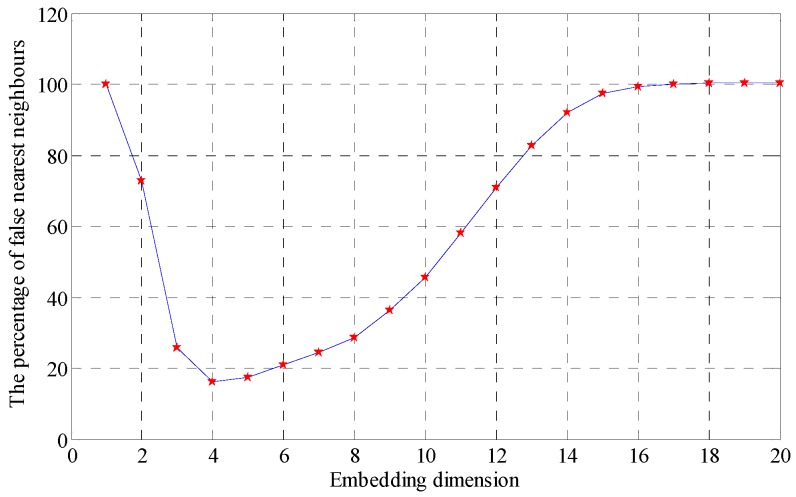
Embedded dimension by the false nearest neighbor point method.

As shown in the Figure 9, the signal is completely surrounded by noise signal, and the signal shock cannot be identified, and the fault characteristic frequency cannot be identified in the envelope spectrum. The signal is calculated with the proposed method, and embedding dimension number is calculated first. The delay time set here is 1, and the maximum embedding dimension number is 20, and the embedding dimension number is obtained for the signal with false nearest neighbor method. The result is as shown in Figure 10.

As shown in the Figure 10, the optimal embedding dimension numbers are 5 or 16. When the embedding dimension number is 5, the SVD obtained after the SV decomposition of reconstructed trajectory matrix is as listed in Table 2.

**Figure 11:**
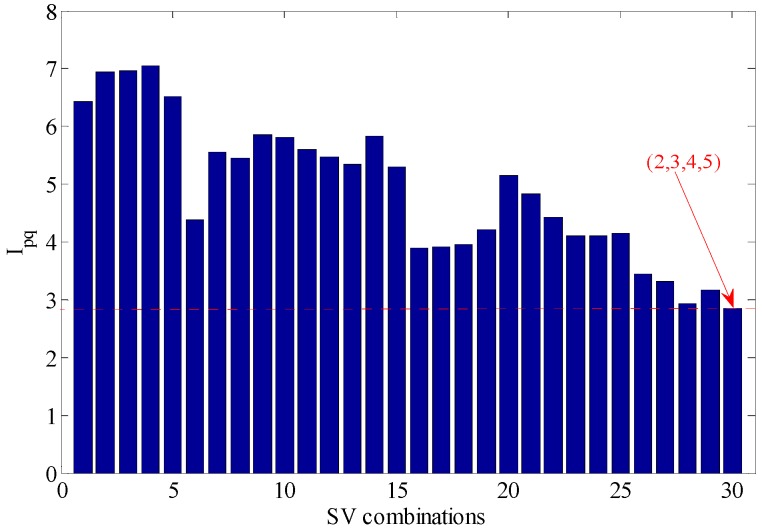
Statistics information criteria value.

Different combinations of feature values are analyzed with an evaluation function of statistical information; the values obtained with the evaluation function of statistical information are as shown in Table 3 and Figure 11.

**Figure 12:**
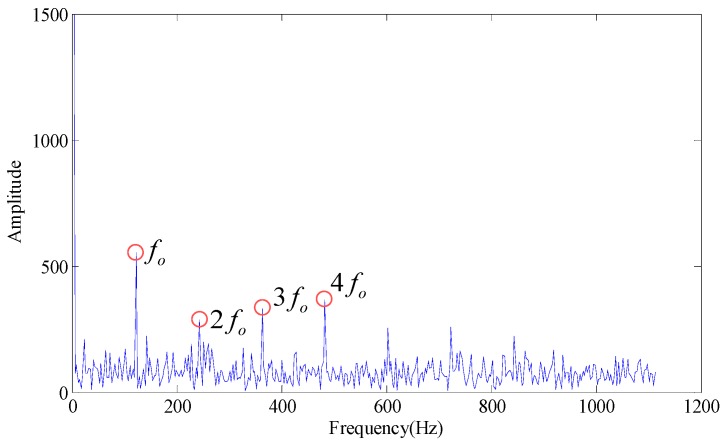
Envelope spectrum (Singular value 1).

According to the Table 3 and Figure 11, when the SV combination is 2, 3, 4 and 5, the value of the evaluation function of statistical information is the minimum, with maximum similarity, therefore, the decomposed signal is turned back into a one-dimensional signal with the rest SV 1 and the envelope spectrum of the restored signal is calculated. As shown in Figure 12, the fault characteristic frequency is obvious and the frequency multiplication can be well distinguished.

In order to verify the status when the embedding dimension number is higher than 5, the status with embedding dimension number of 16 is evaluated. When the embedding dimension number is 16, the data size is very large, and it will be very time consuming if the full combinations are analyzed, therefore, the similarities are calculated with an evaluation function of statistical information for the signals after restoration of each SV and decomposed signal and signals in normal state. The SVs are sequenced from high to low with the values of evaluation function of statistical information. The sequencing results are as shown in Table 4.

**Figure 13:**
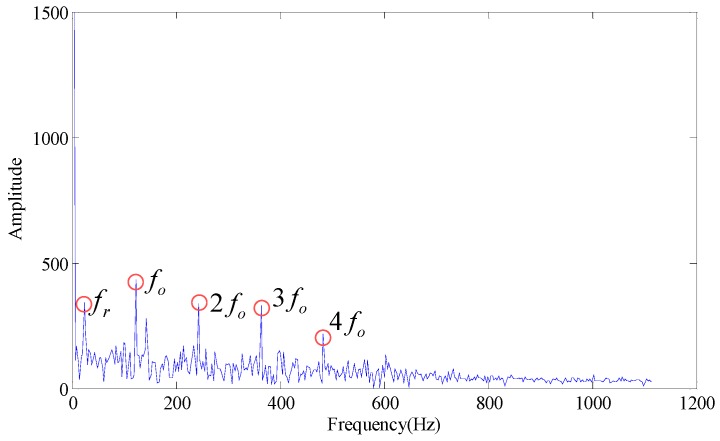
Envelope spectrum (singular values 1, 2, 5).

As shown in Table 4, the SV 1, 2, 3, 5 and 7 are the most similar five values, and all combinations are made with $\lambda_{1},\;\lambda_{2},\;\;\lambda_{3},\;\;\lambda_{5},\;\;\lambda_{7}$; according to the analysis with the one-dimension signal restored from decomposed signal by the combinations and signals in normal state, the energy of the envelope spectrum is the maximum when the SV is 1, 2 and 5, see Figure 13.

### 5.2. Engineering Applications

#### 5.2.1. Experiment Conditions

**Figure 14:**
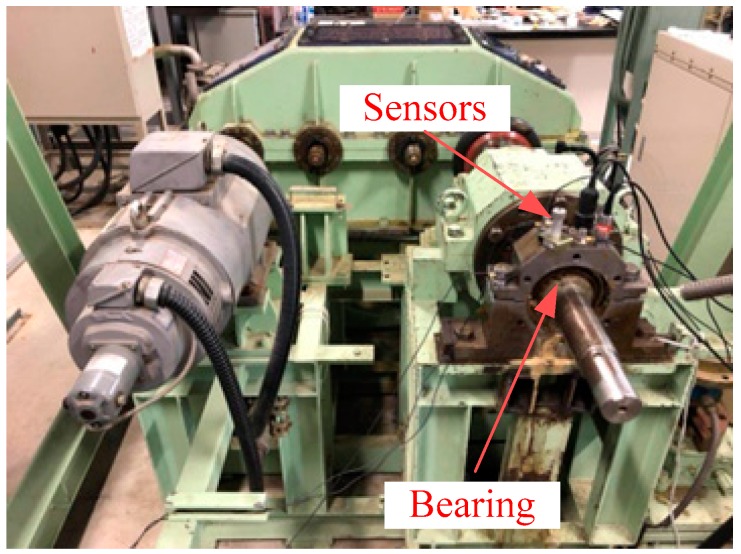
Bearing fault diagnosis experimental system.

In order to verify the effectiveness of the proposed method, an experimental verification is performed with an inner race fault, outer race fault and roller fault under low-speed operation as well as a signal in normal state. Figure 14 shows an experimental platform for a fault diagnosis test.

**Figure 15:**
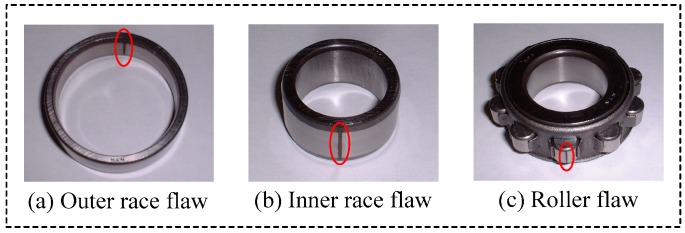
Bearing flaw of outer race, inner race and roller.

The outer race flaw type is N312, and the type of normal bearing, inner race flaw and roller flaw type is NU312, the difference between N312 and NU312 is only the position of the flaw, and other properties are the same. As shown in Figure 15, three different types fault, which are outer race flaw, inner race flaw, and the roller flaw were artificially made by using electro discharge machining, which fault width is 5.0 mm, and depth is 0.03 mm. The original vibration signals of each state were measured by an accelerometer (PCB MA352A60, PCB Piezotronics Inc., New York, NY, USA) with 50,000 Hz sampling frequency. The accelerometer was fixed on the vertical direction of the bearing. While obtaining the vibration signals, the speed of the servo motor was 100 RPM. All the data were stored and recorded and transformed by a collection system that includes a sensor signal conditioner (PCB ICP Model 480C02, PCB Piezotronics Inc.) and a signal recorder (Scope Coder DL750, Yokogawa Co. Ltd., Tokyo, Japan). The fault characteristic frequency at 100 RPM is obtained as shown in Table 5 with the fault characteristic frequency computational formula.

Here, the balls number is 12, contact angle 0, ball diameter 18 mm, inner diameter 87.5 mm, outer diameter 113 mm and RPM 100.

#### 5.2.2. Out Race Fault

**Figure 16:**
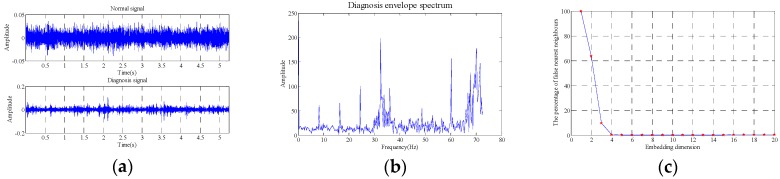
Outer race fault (**a**) time domain waveform, (**b**) envelope spectrum (**c**) embedded dimension.

The data selected is 262,144 (5.24288 s). The comparison of outer race fault and normal state in the time-domain waveform, envelope spectrum and the results obtained with false nearest neighbor (maximum dimension number is 20 and delay time is 1) are as shown in Figure 16.

**Figure 17:**
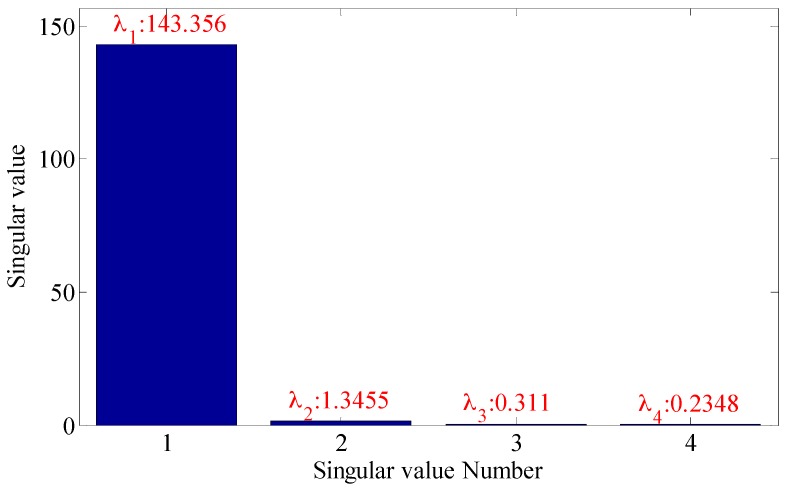
Singular value of trace matrix.

As shown in the Figure 16, the embedding dimension number is optimal at 4, so the embedding dimension number 4 is selected to perform the trajectory matrix reconstruction on the signals and perform SVD on the matrix. The SVs obtained are as shown in Figure 17.

It can be seen that the first two SVs are the largest, and the restored signals and signals in normal state are analyzed with the evaluation function of statistical information with different SV combinations, and the values of evaluation function of statistical information obtained are as shown in Table 6.

**Figure 18:**
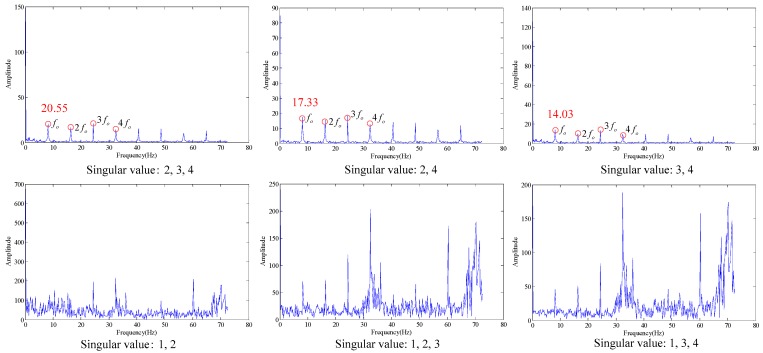
Filtering effective under different singular values.

As shown in the Table 6, when the SV is 1, the similarity is the maximum, and the optimal SV combination at this time is 2, 3 and 4. The verification of its combination is as shown in Figure 18. As shown in the Figure 18, where the three figures can be used to accurately distinguish fault characteristic frequencies, in which the energy corresponding to combination 2, 3 and 4 is the strongest (20.55), and it also has the optimal filtering effect, and the fault is accurately diagnosed.

#### 5.2.3. Roller Fault

**Figure 19:**
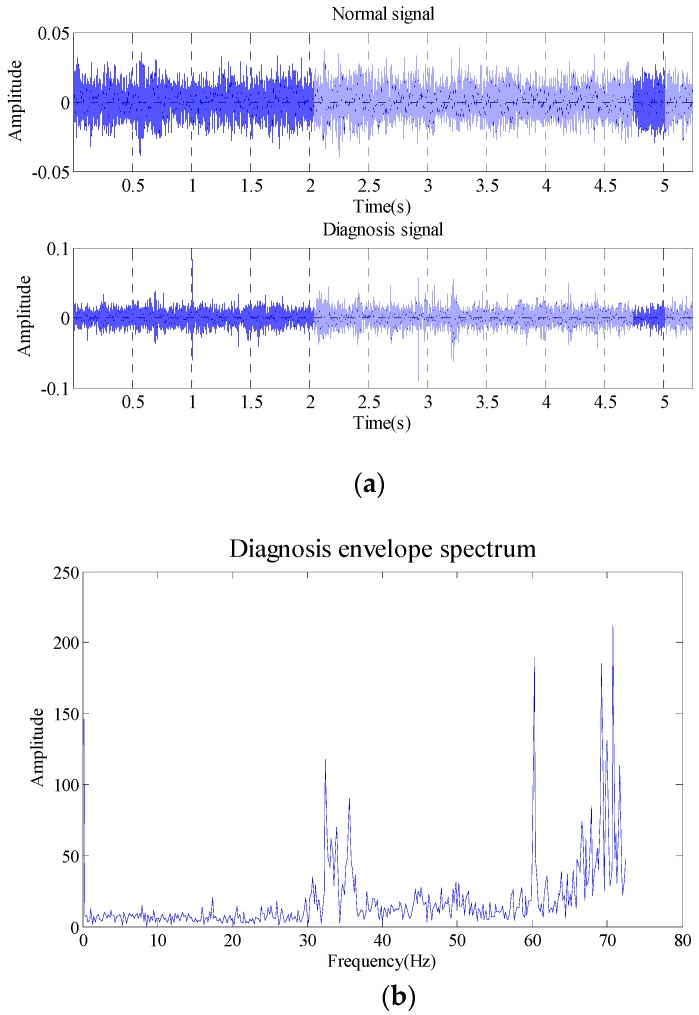

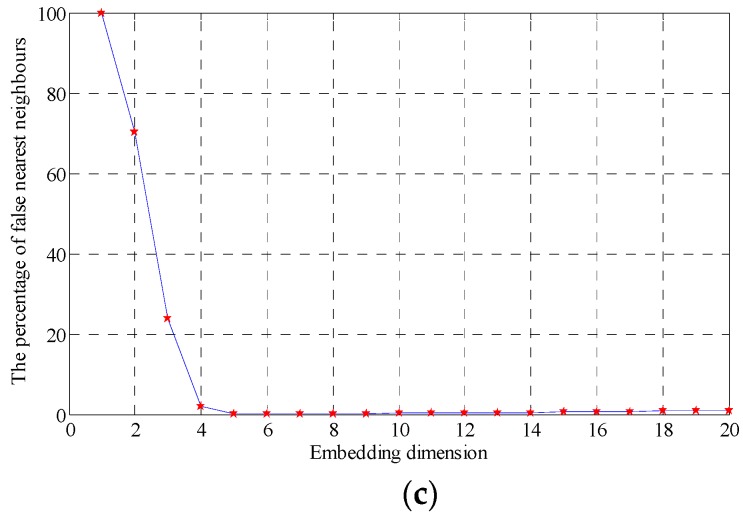
Roller fault (**a**) time domain waveform, (**b**) envelope spectrum (**c**) Embedded dimension.

The data length selected is 262,144 (5.24288 s). The comparisons between roller fault signal and signal in normal state in time-domain waveform, envelope spectrum and the results obtained with false nearest neighbor (maximum dimension number of 20 and delay time of 1) are as shown in Figure 19.

**Figure 20:**
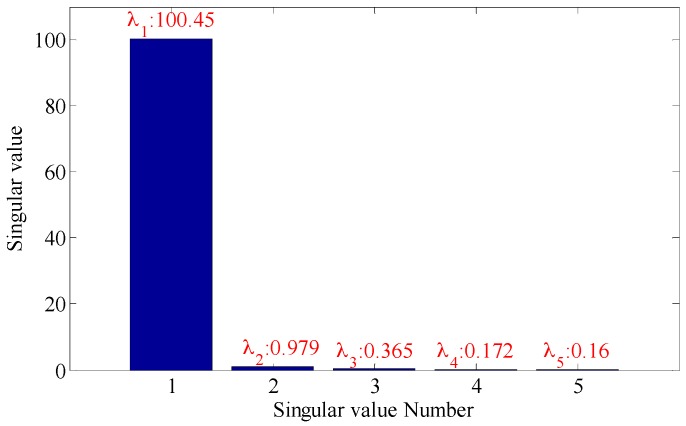
Singular value of the trace matrix.

As shown in the Figure 19, the embedding dimension number is optimal at 5. Trajectory matrix reconstruction and SVD are performed with embedding dimension number of 5. The SVs obtained are as shown in Figure 20.

**Figure 21:**
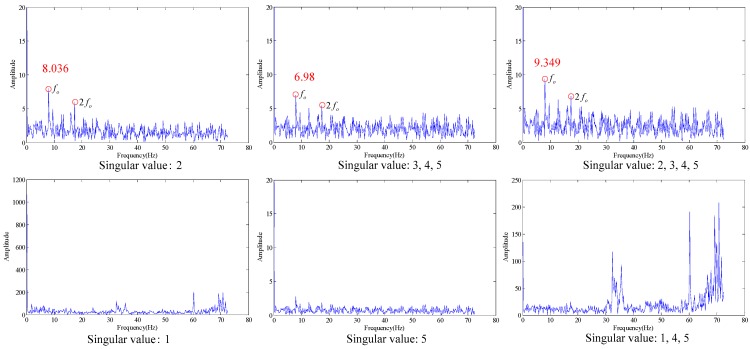
Filtering effect under different singular values.

According to the analysis of evaluation function of statistical information, the optimal SV combination obtained is 2, see Figure 21.

As shown in the Figure 21, the three figures above can all accurately distinguish the fault characteristic frequency, in which the energy is the strongest for the combination that only contains singular value 2 (the first subfigure in Figure 21), also with optimal filtering effect, and the fault is accurately diagnosed at the same time.

#### 5.2.4. Inner Race Fault

**Figure 22:**
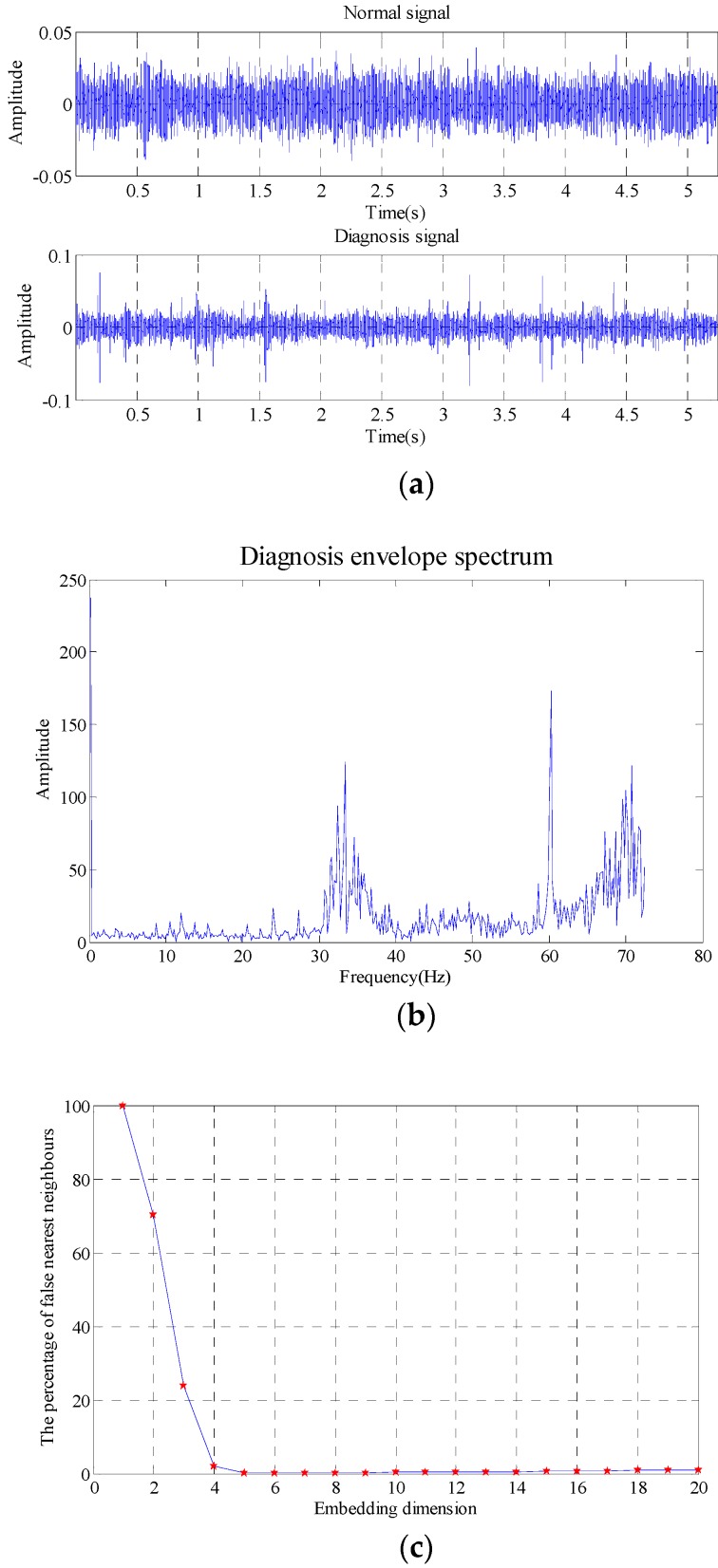
Inner race fault (**a**) time domain waveform; (**b**) envelope spectrum (**c**) embedded dimension.

The data length selected is 262,144 (5.24288 s). The comparison between inner race fault signal and signal in normal state in time-domain waveform, envelope spectrum and the results obtained with false nearest neighbor (maximum dimension number of 20 and delay time of 1) are as shown in Figure 22.

**Figure 23:**
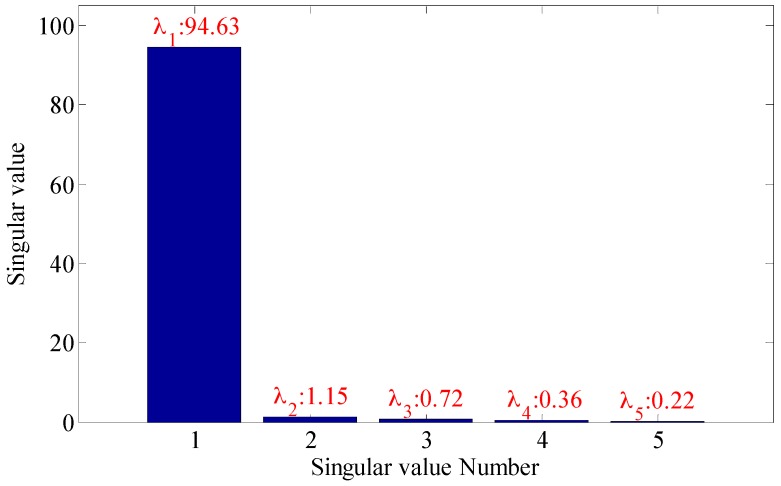
Singular values of the trace matrix.

As shown in the Figure 22, the embedding dimension number is optimal at 5. Trajectory matrix reconstruction and SVD are performed with embedding dimension number of 5. The SVs obtained are as shown in Figure 23.

**Figure 24:**
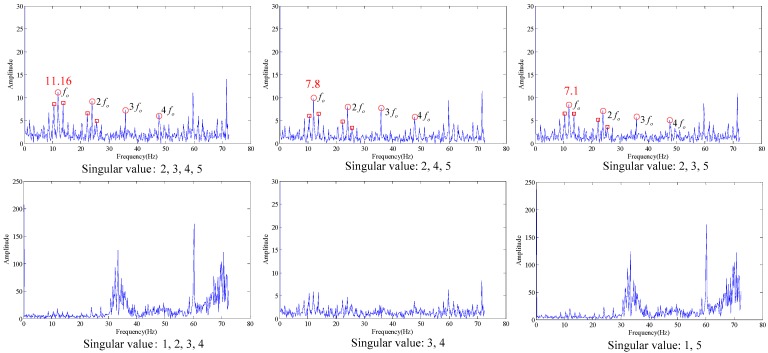
Filtering effective under different singular value.

According to the analysis of statistical information criteria, the optimal SV combinations obtained are 2, 3, 4 and 5. The verification of the effects of the combinations is as shown in Figure 24.

As shown in the Figure 24, the three figures above can all accurately distinguish the fault characteristic frequencies, in which the combination 2, 3, 4 and 5 has the strongest energy, also with optimal filtering effect, and the fault is accurately diagnosed. The detection rate of different bearing health conditions (outer race flaw, inner race flaw and roll flaw) in the experiment is 100%. All of bearing flaws in the experiment can be diagnosed successfully.

### 5.3. Case Western Reserve University Bearing Data

**Figure 25:**
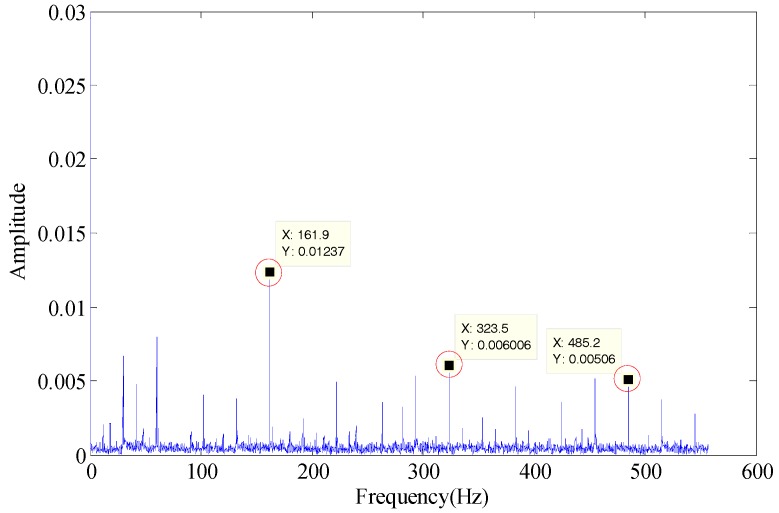
Envelope spectrum of the proposed method.

Case Western Reserve University (CWRU) Bearing Data is a publicly available dataset for bearing fault diagnosis [30]. The details of the data production are available on the website and in [31]. The experiment data number is IR007-0 (inner race flaw), fault depth 2.7940 mm, fault diameter 0.1777mm, motor load 0 and rotating speed 1797 per minute. According to Equation (10), the fault characteristic frequency is 162 Hz. Applying the proposed method, the envelope spectrum is as shown in Figure 25.

As shown in Figure 25, the fault characteristic frequency of the proposed method (161.9 Hz) can be identified and is identical to the calculated result, so the effectiveness of the developed method has been verified successfully.

### 5.4. Comparison with the DCSISE Method

**Figure 26:**
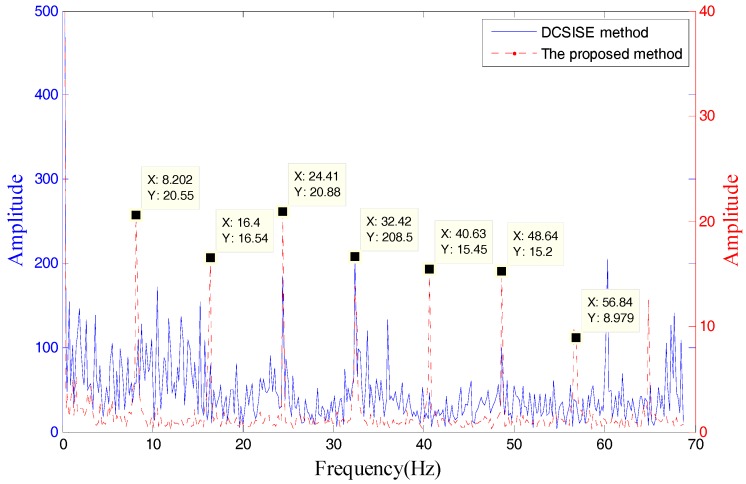
Comparison with the proposed method and DCSISE method.

To verify the advancement of the proposed method, this section compares it with the method published in [20]. The paper used a difference curvature spectrum of incremental singular entropy (DCSISE) to determine the number of effective singular values. The detailed algorithm steps are shown in the reference. The experiment data is the same as Section 5.2.2 (outer race fault). The envelope spectrum of two methods is shown in Figure 26.

As shown in the Figure 26, the proposed method SV combination is 2, 3 and 4, the DCSISE method is 1 and 2. The fault characteristic frequency of the proposed method can be identified, but the DCSISE method can’t identify the fault characteristic frequency, which indicates the proposed method has effectiveness and represents a notable advancement.

### 5.5. Comparison with Fast Kurtogram Method

**Figure 27:**
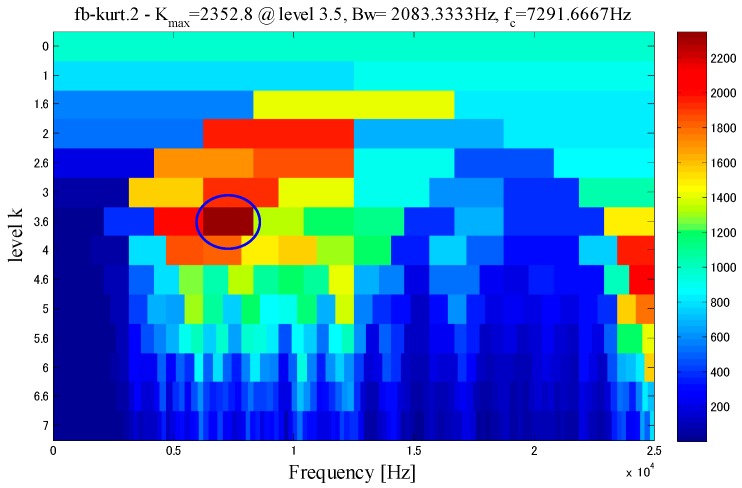
Fast Kurtogram.

In order to furtherly verify the effectiveness of the proposed method, this study compared the inner race at low-speed bearing fault signal with the Fast Kurtogram diagnosis method. The method is described in [32], and the Fast Kurtogram is shown in Figure 27.

**Figure 28:**
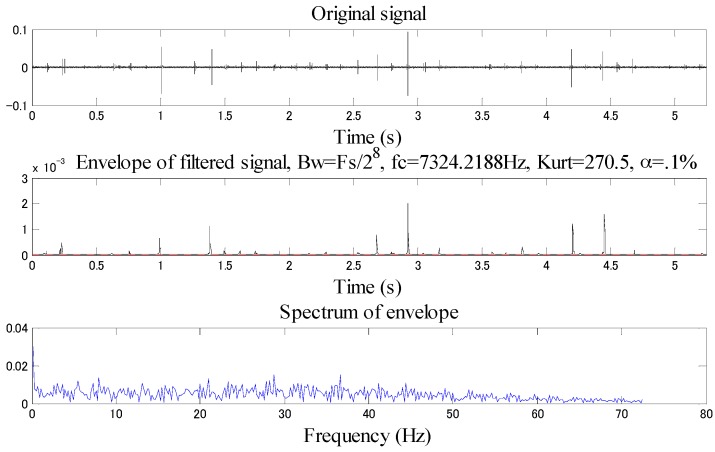
Time domain waveform, envelope, envelope spectrum of the Fast Kurtogram filtered signal.

As shown in the Figure 27, the spectrum kurtosis is the maximum when the central frequency is 7291.6667 Hz, and the bandwidth is 2083.33 Hz. The filtering is performed with such data to obtain the filtered original waveform (time domain), envelope signal and envelope spectrum, see Figure 28.

As shown in Figure 28, the inner race fault characteristic frequency cannot be effectively demodulated with the Fast Kurtogram method, so this method has poor effect in low-speed bearing diagnosis.

## 6. Conclusions

This study proposed an effective SV selection filtering method based on false nearest neighbor and statistical information criteria, and applied it to bearing fault diagnosis. False nearest neighbor can be used to calculate the optimal embedded dimension to reconstruct original signal trajectory matrix. Different SV combinations are selected, and the one-dimension signal restored from SVD signal and signal in normal state are compared, so as to obtain effective SV combinations with statistical information criteria as basis, and this SV combination is the most representative of fault signals. Based on the envelope spectrum method, the signal restored from the optimal SV can be demodulated to effectively analyze fault characteristic frequencies. Experimental verification has been performed for this method through simulation experiments and engineering experiments, which are compared with the DCSISE and Fast Kurtogram methods to demonstrate the fault diagnosis effectiveness of the proposed method against background of strong noise. The SV selection filtering method proposed in the study could be applied further in the fault signal diagnosis of other rotating machinery.

## Figures and Tables

**Table 1:** Singular value of trajectory matrix.

No.	$\lambda_{1}$	$\lambda_{2}$	$\lambda_{3}$	$\lambda_{4}$	$\lambda_{5}$	$\lambda_{6}$	$\lambda_{7}$	$\lambda_{8}$
Singular Value	9,374,507	398,303.5	7884.488	255.2365	12.57116	10.81382	10.46487	4.535536

**Table 2:** Singular values of the trajectory matrix.

No.	$\lambda_{1}$	$\lambda_{2}$	$\lambda_{3}$	$\lambda_{4}$	$\lambda_{5}$
Singular Value	19,942.18	18,697.51	16,517.66	15,683.62	15,296.79

**Table 3:** Statistics information criteria value.

Singular Value	1	2	3	4	5
$I_{pq}$	6.4299	6.9418	6.9639	7.0401	6.5273
Singular Value	1, 2	1, 3	1, 4	1, 5	2, 3
$I_{pq}$	4.3887	5.5665	5.4538	5.8543	5.819
Singular Value	2, 4	2, 5	3, 4	3, 5	4, 5
$I_{pq}$	5.5942	5.4714	5.3488	5.8284	5.2938
Singular Value	1, 2, 3	1, 2, 4	1, 2, 5	1, 3, 4	1, 3, 5
$I_{pq}$	3.8968	3.9179	3.9657	4.2053	5.1614
Singular Value	1, 4, 5	2, 3, 4	2, 3, 5	2, 4, 5	3, 4, 5
$I_{pq}$	4.8399	4.4298	4.0966	4.114	4.1387
Singular Value	1, 2, 3, 4	1, 2, 3, 5	1, 2, 4, 5	1, 3, 4, 5	2, 3, 4, 5
$I_{pq}$	3.4388	3.313	2.9331	3.1753	2.8386

**Table 4:** SVs are listed in ascending order based on statistical information.

**No.**	**1**	**2**	**3**	**4**	**5**	**6**	**7**	**8**
Singular Value	$\lambda_{1}$	$\lambda_{2}$	$\lambda_{3}$	$\lambda_{5}$	$\lambda_{7}$	$\lambda_{4}$	$\lambda_{6}$	$\lambda_{8}$
$I_{pq}$	2.0746	2.1745	2.3433	2.5632	2.8324	3.1023	3.3723	4.2113
**No.**	**9**	**10**	**11**	**12**	**13**	**14**	**15**	**16**
Singular Value	$\lambda_{9}$	$\lambda_{10}$	$\lambda_{11}$	$\lambda_{12}$	$\lambda_{13}$	$\lambda_{14}$	$\lambda_{15}$	$\lambda_{16}$
$I_{pq}$	4.2452	4.2514	4.8346	5.2146	5.7246	5.8532	6.0123	6.3468

**Table 5:** Fault characteristic frequency of inner race fault, outer race fault and roller fault.

Fault	Fault Characteristic Frequency
Outer Race Flaw	8.00 Hz
Inner Race Flaw	11.9 Hz
Roller Flaw	8.02 Hz

**Table 6:** Statistics information criteria value.

Singular Value	1	2	3	4	1, 2
$I_{pq}$	11.6379	80.4025	101.7774	118.157	11.7015
Singular Value	1, 3	1, 4	2, 3	2, 4	3, 4
$I_{pq}$	11.6448	11.6845	93.3342	109.9708	46.8683
Singular Value	1, 2, 3	1, 2, 4	1, 3, 4	2, 3, 4	
$I_{pq}$	11.722	11.7602	11.6945	180.0193	
